# Advancing biology through a deeper understanding of zebrafish ecology and evolution

**DOI:** 10.7554/eLife.05635

**Published:** 2015-03-25

**Authors:** David M Parichy

**Affiliations:** Department of Biology, University of Washington, Seattle, United States

**Keywords:** the natural history of model organisms, pigment pattern, sex determination, behavior, population genetics, phylogeny, zebrafish

## Abstract

Over the last two decades, the zebrafish has joined the ranks of premier model organisms for biomedical research, with a full suite of tools and genomic resources. Yet we still know comparatively little about its natural history. Here I review what is known about the natural history of the zebrafish, where significant gaps in our knowledge remain, and how a fuller appreciation of this organism's ecology and behavior, population genetics, and phylogeny can inform a variety of research endeavors.

**DOI:** http://dx.doi.org/10.7554/eLife.05635.001

## Introduction

Like so many model organisms, the zebrafish was chosen for its tractability and, especially, its potential for genetic analysis and cellular observation. The founder of ‘modern’ zebrafish research, George Streisinger, had a passion for fish but it seems unlikely that he chose this particular minnow because of any prior insights into its natural history or its phylogenetic position within the teleosts (bony fishes). Rather, the zebrafish was readily available, it was easy to breed, and its lovely, transparent embryo was quick to develop. Streisinger's efforts at the University of Oregon, and the hard work of many early adopters of the species, most notably other labs in Oregon, Tübingen and Boston, propelled zebrafish into the top tier of NIH-funded biomedical models (Grunwald and Eisen, 2002; Kinth et al., 2013). The zebrafish model ‘system’ now comprises a sequenced genome, thousands of mutants, transgenic tools, staging series, and a wealth of know-how for imaging, embryological manipulation, drug discovery and more.

Given all of these resources, one might wonder how much is known about zebrafish as an organism (rather than as a system), and whether it matters. Here, I review briefly what we do and don't know about wild zebrafish, and reflect upon the ways in which a deeper appreciation of zebrafish in their natural habitat can inform a range of biological enquiries.

## Range and habitat

**Figure 1.**
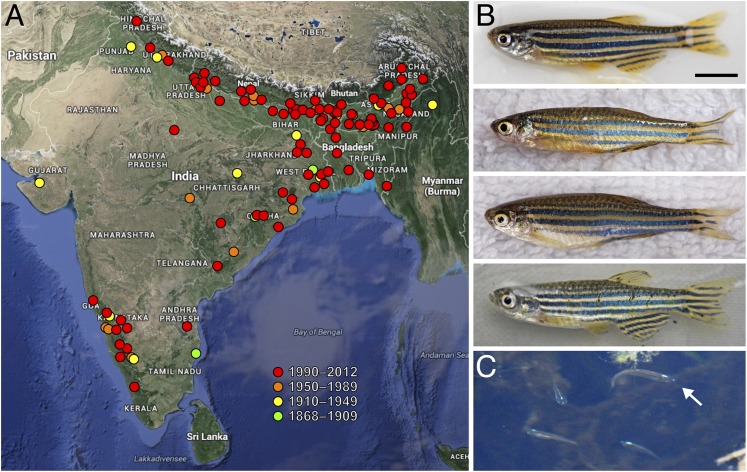
Zebrafish and their geographic range. (**A**) Historic and more recent sites where zebrafish have been reported in India, Nepal, Bangladesh and possibly Myanmar (Spence et al., 2006; Engeszer et al., 2007b; Spence et al., 2008; Whiteley et al., 2011; Arunachalam et al., 2013). (**B**) Zebrafish from several populations in northeastern India (Engeszer et al., 2007b). The upper two fish are males and the lower two fish are females; males tend to have a slightly yellow cast ventrally. (**C**) A group of zebrafish (a single fish is highlighted with the arrow) in a stream-side pool in Meghalaya, India, north of Bangladesh. Scale bar: 5 mm (**B**). Image credits: D Parichy. **DOI:** http://dx.doi.org/10.7554/eLife.05635.002

Zebrafish were described by a Scottish physician, Francis Hamilton, under the auspices of the British East India Company (Hamilton, 1822). Hamilton’s “beautiful fish” with its “several blue and silver stripes on each side” has undergone a few changes to its Latin name and is now correctly referred to as *Danio rerio* (original and more recent synonyms include *Cyprinus* and *Brachydanio*). Formally described in the state of Bihar in northeastern India, zebrafish have also been collected in the south and west of peninsular India, past the city of Bangalore, and beyond India, as far north as Pakistan and Nepal, as well as east into Bangladesh and possibly Myanmar (Figure 1) (Engeszer et al., 2007b; Spence et al., 2008; Arunachalam et al., 2013).

**Figure 2.**
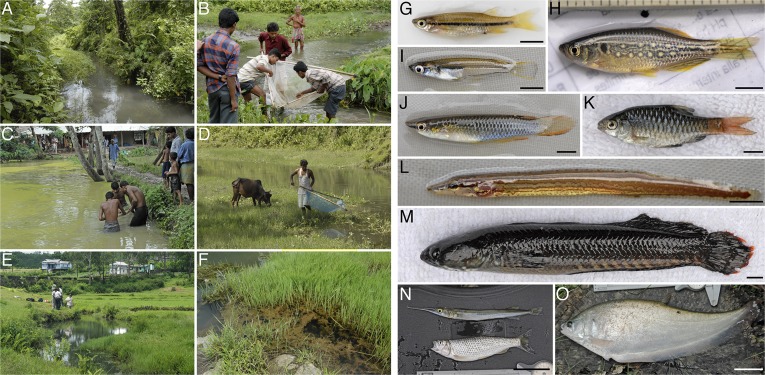
Zebrafish habitat and co-occurring species. (**A**–**F**) Zebrafish are found in streams both pristine (**A**) and shared with people (**B**, **D**, **E**), in ephemeral pools at stream margins (**F**; a close-up of Figure 1C), and in man-made bodies of water (**C**). (**G**–**K**) Many fish might compete with zebrafish at one or more of its life stages, including *E. danricus* (**G**), *D. dangila* (juvenile; **H**), *Oryzias* (**I**), *Aplocheilus panchax* (**J**), and *P. shalynius* (**K**). (**L**–**O**) Among potential predators are *Mastecembalus* (**L**), *Channa* (**M**), *Xenentodon* (top) and *Barilius* (**N**), and *Notopterus* (**O**). For details see Engeszer et al. (2007b). Scale bars: 5 mm (**G**–**M**); 5 cm (**N**, **O**). Image credits: D Parichy. **DOI:** http://dx.doi.org/10.7554/eLife.05635.003

Although found by Hamilton near the Ganges, it seems unlikely that individual zebrafish ever intentionally end up in such a large river. Rather, their typical habitat consists of shallow, slow moving streams, and, particularly, still pools that form alongside streams during the monsoons. The water in these streams and pools is typically clear, but there can be mud, sand or gravel on the bottom of the stream or pool, so the water often becomes turbid in the rain. Some habitat complexity is provided by aquatic vegetation, and cover is sometimes available from overhanging vegetation or from overhanging banks. Having co-occurred with humans for thousands of years, zebrafish also make themselves at home in rice paddies, drainage ditches, stock ponds and the like, although they certainly suffer the effects of pollution and habitat loss as well (Figure 2A–F). Zebrafish have been reported at elevations of ∼8–1576 m and in a range of water conditions, including temperatures between 12–39°C, pH levels of 5.9–9.8, and salinities of ∼0.01–0.8 (Spence et al., 2006; Engeszer et al., 2007b; Arunachalam et al., 2013).

Zebrafish are omnivores, consuming larval and adult insects, as well as small crustaceans and other zooplankton, but also partaking of algae, plant material and assorted detritus (McClure et al., 2006; Spence et al., 2007b; Arunachalam et al., 2013). Interestingly, recently caught wild zebrafish and domesticated lab strains have similar intestinal bacteria, suggesting a core gut microbiota (Roeselers et al., 2011) important for growth and development (Cheesman et al., 2011; Semova et al., 2012).

Potential competitors for food, and perhaps other resources as well, include other minnows, like *Esomus* and *Puntius*, and similar small fishes (Figure 2G,I–K). Zebrafish can also be found with larger *Danio* species (Figure 2H); although adults may occupy distinct microhabitats, competition among larvae is conceivable. Of course, zebrafish are certainly on the menu themselves: snakehead fish (*Channa*), knifefish (*Notopterus*) and catfish, birds such as kingfishers and herons, and even dragonfly larvae, are all likely predators of adults, and a great many species probably eat zebrafish eggs and larvae (Figure 2L–O; Engeszer et al., 2007b). Nevertheless, the impact of competition and predation on the survival and reproduction of wild zebrafish remain entirely unknown.

## Behavioral interactions and syndromes

Field observations of zebrafish behavior are few and anecdotal, and so much of what zebrafish do in nature has to be inferred from their behavior in the lab. One behavior that has received considerable attention is the formation of loose social aggregations, or shoals, which have been observed in the field (see Figure 1C) and studied in the lab (Engeszer et al., 2007b; Gerlai, 2014). This behavior might provide protection from predators, improved foraging success, or access to mates. Shoaling increases steadily from early larval stages, and individuals ‘imprint’ on a particular visual phenotype, showing a preference for this phenotype by the time they are juveniles (Engeszer et al., 2004, 2007a; Spence and Smith, 2007; Mahabir et al., 2013). Interestingly, wild-caught and lab fish (both previously imprinted on the ‘wild type’) have similar preferences for prospective shoaling partners when presented with fish that have different pigment patterns and other phenotypes, although the specifics differ between sexes: female preferences appear to be complex, whereas males show strong preferences that correlate with stripe quality and species identity (Engeszer et al., 2008). Many additional factors might also influence whether or not zebrafish shoal together in the wild, including fish size, group size, sex ratio, olfactory stimuli, kin recognition, predation risk and light regime (e.g., Pritchard et al., 2001; Gerlach and Lysiak, 2006; Ruhl et al., 2009).

Lab strains of zebrafish spawn all year round, but breeding in the wild occurs primarily during the summer monsoons, when ephemeral pools appear; these presumably offer plenty to eat and some shelter from currents and predators. Still waters might also facilitate pheromonal communication relevant to oogenesis and courtship (Bloom and Perlmutter, 1977; van den Hurk and Lambert, 1983; van den Hurk et al., 1987; Gerlach, 2006). Spawning tends to occur near daybreak, and can involve male territoriality, as well as female preferences for oviposition (egg-laying) sites (Spence et al., 2007a, 2008). Lab studies indicate that courtship and mating behaviors are stereotypic, although some of the details may depend on the conditions in which observations have been made. Behaviors include the initial approach; chasing by the male and touching of the male's nose to the female's side or tail; male circling and quivering; the female leading the male to an oviposition site, or the male pinning the female against an object; and oviposition itself (Darrow and Harris, 2004; Sessa et al., 2008; Kang et al., 2013). Females can lay up to several hundred eggs at once, or smaller numbers every few days, but the actual number of offspring from any given spawning is highly variable. Indeed, males can differ in the clutch sizes they elicit from females, (Spence and Smith, 2006), possibly owing to differences in body size (Skinner and Watt, 2007); dominance hierarchies can also influence reproductive success (Paull et al., 2010). Although reproductive maturity can be reached in as little as 4–6 weeks in the lab, where zebrafish are known to live for up to several years, we don't as yet know about the timing of their maturation or their longevity in the wild. A deeper understanding of courtship and breeding preferences, as well as life history in nature, will be interesting, and may facilitate research in the lab through improvements in spawning and rearing efficiencies (Sessa et al., 2008; Adatto et al., 2011; Nasiadka and Clark, 2012).

Recently, wild zebrafish brought to the lab have provided new insights into behavioral syndromes, in which behaviors co-vary, as in a continuum of boldness and aggression, or correlated changes that occur during domestication (for example, changes in both fearfulness and activity patterns) that likely derive from intentional selection on some traits and relaxed selection on others (Moretz et al., 2007; Norton et al., 2011). Including wild zebrafish in such studies dramatically expands the range of variation. Indeed, comparisons of zebrafish isolated from different geographic regions, and different lab strains, have revealed striking differences in behavioral syndromes among populations (Robison and Rowland, 2005; Oswald and Robison, 2008; Drew et al., 2012; Martins and Bhat, 2014). That such differences can be heritable (Wright et al., 2006; Oswald et al., 2013) suggests that the genetic bases for natural variation in behavioral syndromes, and the evolution of behavioral traits more generally, can be studied using this species. Of critical importance to all of these endeavors are additional observations and experiments in the field, in order to better understand the zebrafish behavioral repertoire and its significance for individual fitness, and also to determine the extent to which habitat differences between field and lab might impact our ability to generalize results from one context to the other.

## Population genetics and sex determination

Genomics is another arena in which wild zebrafish are providing valuable insights. Most lab zebrafish represent any of several commonly used strains initiated with founders obtained from the pet trade or, in some cases, from collections in the wild. Some strains have been maintained to preserve allelic variation, others to intentionally minimize genetic diversity, to facilitate genetic mapping and genome editing, and to control genetic background effects on mutant and other phenotypes (Haffter et al., 1996; Rauch et al., 1997; Trevarrow and Robison, 2004; LaFave et al., 2014). One theme to emerge is that laboratory strains differ substantially from one another, and even some of the more ‘inbred’ strains maintain remarkably high levels of genetic diversity, as measured by microsatellite variation, single nucleotide polymorphisms (SNPs), and gene copy number variants (Nechiporuk et al., 1999; Guryev et al., 2006; Coe et al., 2009; Whiteley et al., 2011; Brown et al., 2012).

When samples from wild populations are analyzed, it becomes clear that even the extensive variation in lab strains represents but a tiny fraction of total zebrafish genetic diversity (Coe et al., 2009; Whiteley et al., 2011; Brown et al., 2012; Patowary et al., 2013). Moreover, despite the predilection of zebrafish to occupy flood plains—which might suggest extensive gene flow—analyses of wild fish across the species' range indicate this is not always the case: several populations in the Ganges/Brahmaputra River basins form a genetic group (into which also fall three lab strains), but two other, deeply divergent groups have been identified as well (Whiteley et al., 2011). It seems likely that further population-level sampling will reveal additional, genetically differentiated populations, which could provide outstanding opportunities to understand local adaptation.

The considerable genetic diversity of zebrafish is put into perspective by comparisons with the human genome. For instance, complete genomic sequences of just two gynogenetic (’double haploid‘; Streisinger et al., 1981) zebrafish of different lab strains revealed ~7 million SNPs between them; a single, wild zebrafish harbored over 5 million SNPs within its own genome (Howe et al., 2013; Patowary et al., 2013). By contrast, more than a thousand sequenced human genomes have yielded a ‘mere’ 38 million SNPs in total and an average of only 3.6 million SNPs per diploid individual (1000 Genomes Project Consortium et al., 2012). If the above zebrafish results are typical of this species, this works out to SNPs being ∼four-fold more frequent in zebrafish than they are in humans, after correcting for genome size. Copy number variants are likewise ∼1.5-fold more prevalent in zebrafish than in human genomes (Brown et al., 2012). It remains unclear why zebrafish are so diverse genetically; wild zebrafish do not seem to carry an excess of lethal mutations as compared to other vertebrates (McCune et al., 2002, 2004).

This level of genetic variation in zebrafish poses some challenges, such as the added complexity of assembling genomic sequence and the need to control rigorously for genetic background in experiments. It also provides opportunities: to study genome evolution at a fine scale, and gene effects that are relevant to complex traits and genetic disease in admixed human genomes. Benefitting recently from this diversity is our understanding of sex determination. In comparison to so many species—biomedical models and otherwise—the lack of a demonstrated sex-determining system had long been perplexing, and just a bit embarrassing, to researchers using zebrafish (though presumably not to the fish themselves). Genetic analyses of lab strains have identified chromosomal regions associated with sex determination, yet, surprisingly, these differed between studies (Bradley et al., 2011; Anderson et al., 2012; Liew et al., 2012; Howe et al., 2013). Analyses of wild zebrafish suggest a reason for the discrepancies: these fish have a major sex determinant (WZ/ZZ) on chromosome 4—which has features similar to sex chromosomes in other species—yet this determinant has been lost from lab strains (Wilson et al., 2014). This suggests that founder effects, or domestication itself, led to seemingly ad hoc systems employing multiple sex determinants, probably of small original effect in the wild. Thus, comparison of wild and lab zebrafish has revealed a serendipitous example of how sex-determining mechanisms can evolve. Whether this discovery has implications for understanding reproductive behavior or physiology in lab strains relative to wild fish has yet to be explored.

## Zebrafish relatives and their phylogeny

**Figure 3.**
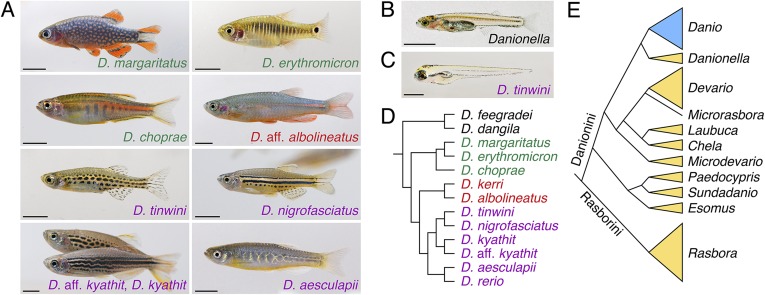
Zebrafish relatives and phylogeny. (**A**) Examples of *Danio* pigment patterns, including spotted and striped morphs of *D. kyathit*. (**B**) Adult male of the miniaturized species *Danionella dracula*. (**C**) Larval *D. tinwini* (3 days post-fertilization), illustrating a typical *Danio* early larval pigment pattern. (**D**) Relationships within *Danio* (redrawn from: McCluskey and Postlethwait, 2015). (**E**) Relationships within Danioninae (simplified and redrawn from: Tang et al., 2010). Branch lengths in **D** and **E** are arbitrary. Scale bars: 5 mm (**A**, **B**); 0.5 mm (**C**). Image credits: D Parichy. **DOI:** http://dx.doi.org/10.7554/eLife.05635.004

Because every organism is a mix of shared and derived traits, even the biomedical ‘models’ sometimes differ markedly from other species in their broader phylogenetic group. Comparisons with ‘non-model’ relatives can thus provide important insights into the generality of inferences about development, genetics, and behavior (Parichy, 2005; Harris et al., 2014). Of course, diversity of form and the evolution of underlying mechanisms can be interesting in their own right. Although zebrafish of disparate populations are not grossly dissimilar morphologically (Arunachalam et al., 2013) (see for example, Figure 1B), other species in the large zebrafish family Cyprinidae differ dramatically in size, shape and other traits (Tang et al., 2010). Indeed, the subfamily Danioninae includes not only zebrafish, which grows to 4–5 cm, but also *Danio dangila* (Figure 2H), which grows to ∼13 cm, and some of the world's smallest vertebrates, like *Danionella* (Figure 3B) and *Paedocypris*, which mature in a larval-like form at only ∼1–1.5 cm (Roberts, 1986; Britz et al., 2009; Mayden and Chen, 2010).

One trait that differs among *Danio* and that has received attention is the pigment patterns of adult fish. In contrast to the distinct stripes of zebrafish, other danios have vertical bars, spots, reduced numbers of stripes, uniform arrangements of pigment cells, and other patterns (Figure 3A) (McClure, 1999; Parichy and Johnson, 2001; Quigley et al., 2004, 2005; Parichy, 2006). When zebrafish are crossed to other *Danio* species in the lab, the hybrid progeny's pigmentation patterns often resemble that of zebrafish, indicating that zebrafish pigmentation alleles are frequently dominant to those of other species (Parichy and Johnson, 2001; Quigley et al., 2005). A pattern consisting initially of two stripes is likely to be ancestral in *Danio*, and vestiges of these stripes occur in many species, even ones that ultimately develop very different patterns (Quigley et al., 2004; Mills et al., 2007). In zebrafish, adult stripes comprise pigment cells of several lineages (Parichy and Spiewak, 2015) and the formation of this pattern depends on thyroid hormone (McMenamin et al., 2014), positional cues, and interactions among the pigment cells themselves (Nakamasu et al., 2009; Frohnhofer et al., 2013; Patterson and Parichy, 2013; Watanabe and Kondo, 2015). Across *Danio*, the contributions of pigment cell classes to adult patterns differ, as do the genetic requirements of the cells (Quigley et al., 2004; McMenamin et al., 2014) and probably the nature of pigment cell interactions as well (Quigley et al., 2005). A recent study identified *cis* regulatory changes in gene expression—and associated alterations in the timing of pigment cell differentiation—that have likely contributed to the evolution of a uniform pattern in *D. albolineatus* (Patterson et al., 2014).

Although the molecular and cellular mechanisms that underlie pattern development and evolution are becoming better understood, the behavioral and ecological significance of *Danio* pigment patterns remain largely unknown. By extension, it remains unclear whether species differences in patterns are themselves adaptive; alternative phenotypes might simply represent independent and equally good ‘solutions’ to similar selective factors. Adult pigment patterns of other teleosts can function in species recognition, mate choice, and predation avoidance (Price et al., 2008), and stripes of adult zebrafish seem likely to influence shoaling (Engeszer et al., 2008). A fuller understanding of pattern significance will require not only more experiments, but also more information about the differences in species' habitats, including factors both biotic (such as predation regimes) and abiotic (such as light quality).

In contrast to the diversity of adult patterns, early larval patterns of different *Danio* species are nearly indistinguishable from one another (Quigley et al., 2004) (Figure 3C). Because larvae develop in shallow water, and larval pigmentation covers the central nervous system and developing gonads, protection from UV exposure (eg. Mueller and Neuhauss, 2014) is an attractive, albeit untested, functional hypothesis to explain the evolutionary conservation of this pattern.

Analyses of trait evolution and mechanisms of speciation are greatly facilitated by a robust understanding of species relationships. To date, studies of the subfamily Danioninae have arrived at somewhat different interpretations of the group (Fang, 2003; Rüber et al., 2007; Fang et al., 2009; Mayden and Chen, 2010; Liao et al., 2011), although these differences are likely to be resolved with sampling of additional *Danio* species and more sequence within species. A recent phylogenetic analysis suggests the existence of two large ‘tribes’ within Danioninae, Rasborini and Danionini (Figure 3E), the latter including *Danio* and *Danionella*, as well as *Esomus* and *Devario* (such as the ‘giant danio’ *Devario aequipinnatus*, common in the pet trade) (Tang et al., 2010). Interestingly, this phylogenetic analysis also suggests that extreme miniaturization may have evolved independently in *Danionella* and *Paedocypris*.

Within *Danio* itself, 20 species are considered valid taxonomically (Froese and Binohlan, 2014) but several have yet to be included in phylogenetic reconstructions [e.g., (Fang, 1997a, 1997b, 2000; Fang and Kottelat, 1999; Kullander et al., 2009; Kullander, 2012)]. Many species also likely await discovery, particularly in Myanmar and nearby countries, which seem to be the center of *Danio* diversity.

Phylogenetic reconstructions of *Danio* mostly agree that two larger species, *D. dangila* and *D. feegradei*, split early from the other, smaller species, indicating dramatic evolutionary changes in body size even within this genus. Phylogenies also agree on several internal groupings, although the precise ordering of species closest to zebrafish has been unclear (Fang, 2003; Quigley et al., 2005; Mayden et al., 2007; Fang et al., 2009). A recent study using extensive genomic sampling (McCluskey and Postlethwait, 2015) to examine relationships within a ‘*D. rerio* species group’ identified the poorly known *D. aesculapii* (Kullander and Fang, 2009) as a candidate sister species to zebrafish (Figure 3A,D). Interestingly, most of these species are restricted to one or two hydrologic basins; the two species with the broadest ranges, *D. rerio* and *D. albolineatus*, are non-overlapping, whereas *D. rerio* shares a basin in the east of its range with *D. aesculapii*. This same study also revealed instances of gene flow during the origin of these species, including the transfer of alleles between *D. kyathit* and zebrafish lineages, and between *D. aesculapii* and *D. kerri*/*D. albolineatus* lineages. Such ‘horizontal’ movements of alleles complicate the assessment of species relationships: indeed, analyses that allowed for different gene trees across loci (reflecting ancestral instances of hybridization and genetic introgression) provided only weak support for the sister relationship between *D. rerio* and *D. aesculapii*. It will be exciting to further unravel how speciation is proceeding in this group as new *Danio* are discovered and new sequences gathered for analysis, and as roles for hybridization, as well as for geography and other potential isolating mechanisms, are defined.

## Future directions

Recent studies illustrate how wild zebrafish and its relatives can contribute to research programs spanning ecology and behavior, genetics and genomics, and development and evolution. Some specific topics that would benefit from more attention have been cited already and some particularly compelling open questions are listed in Box 1. An additional fruitful area will be the development of genomic resources, including fully sequenced genomes for other species of *Danio* and for more distant cyprinids. These additional genomes will greatly facilitate the identification of gene regulatory domains and how they evolve (Müller et al., 2002; Camp et al., 2012; Patterson et al., 2014) and will provide new insights into the evolution of genes, genomes and phenotypes more broadly.

10.7554/eLife.05635.005

Box 1. **Outstanding questions about the natural history of the zebrafish**

- What are the selective factors (biotic and abiotic) that impinge upon zebrafish survival and reproduction in the wild?
- Are there subtle—or not so subtle—differences in morphology, physiology or behavior among natural populations, and are such differences adaptive?
- Do female zebrafish choose their mates in nature, and if so, what criteria do they use?
- What are the specific genetic changes underlying the evolution of divergent sex determination mechanisms between wild fish and lab strains?
- How many more *Danio* species are there, what are their evolutionary relationships and what factors have influenced speciation and morphological diversification in the genus?

**DOI:** http://dx.doi.org/10.7554/eLife.05635.005

The differences between wild and lab zebrafish cited above also serve as a cautionary tale about generalizing from particular populations to the species as whole. For example, given their range and genetic variability, a single set of optimal conditions for the growth and development of wild zebrafish is unlikely, let alone for lab strains that have experienced very different selective regimes. Likewise, it remains unclear whether environmental enrichment, to mimic the habitat complexity that wild fish can experience, also benefits lab strains, particularly when such interventions are balanced against costs, such as the increased difficulty of observing fish and the increased accumulation of detritus, which themselves can lead to morbidity and mortality. What is clear is that a deeper understanding of zebrafish natural history will benefit the health and well being of fish in the lab, as well as research productivity, when combined with a holistic view of variation in behavior, genetics, and the broader goals of lab research. The promise of integrating a deeper understanding of zebrafish ‘the organism’ with zebrafish ‘the system’ suggests exciting times to come for the devotees of Hamilton's beautiful fish.
